# Protocol for a randomised controlled feasibility trial of exercise rehabilitation for people with postural tachycardia syndrome: the PULSE study

**DOI:** 10.1186/s40814-020-00702-1

**Published:** 2020-10-19

**Authors:** Gordon McGregor, Siew Wan Hee, Helen Eftekhari, Nikki Holliday, Gemma Pearce, Harbinder Sandhu, Jane Simmonds, Shivam Joshi, Lesley Kavi, Julie Bruce, Sandeep Panikker, Boon Lim, Sajad Hayat

**Affiliations:** 1 Department of Cardiopulmonary Rehabilitation, Centre for Exercise & Health, University Hospitals Coventry & Warwickshire NHS Trust, Watch Close, Coventry, CV1 3LN UK; 2 Centre for Sport, Exercise and Life Sciences, Coventry University, Coventry, UK; 3 Warwick Clinical Trials Unit, Warwick Medical School, University of Warwick, Coventry, UK; 4 Division of Health Sciences, Warwick Medical School, University of Warwick, Coventry, UK; 5 Department of Cardiology, University Hospitals Coventry & Warwickshire NHS Trust, Coventry, UK; 6 UCL Great Ormond Street Institute of Child Health, Faculty of Population Health, University College London, London, UK; 7 Research & Development, University Hospitals Coventry & Warwickshire NHS Trust, Coventry, UK; 8 POTS UK, Birmingham, UK; 9 Department of Cardiology, Imperial College Healthcare NHS Trust, London, UK; 10 Department of Cardiology, Hamad Medical Corporation, Doha, Qatar

**Keywords:** Postural orthostatic tachycardia syndrome, Exercise, Rehabilitation, Dysautonomia, Cardiac rehabilitation, Randomised controlled trial, Complex intervention, Feasibility

## Abstract

**Background:**

Postural orthostatic tachycardia syndrome (POTS) is an autonomic nervous system disorder causing an abnormal cardiovascular response to upright posture. It affects around 0.2% of the population, most commonly women aged 13 to 50 years. POTS can be debilitating; prolonged episodes of pre-syncope and fatigue can severely affect activities of daily living and health-related quality of life (HRQoL). Medical treatment is limited and not supported by randomised controlled trial (RCT) evidence. Lifestyle interventions are first-line treatment, including increased fluid and salt intake, compression tights and isometric counter-pressure manoeuvres to prevent fainting. Observational studies and small RCTs suggest exercise training may improve symptoms and HRQoL in POTS, but evidence quality is low.

**Methods:**

Sixty-two people (aged 18–40 years) with a confirmed diagnosis of POTS will be invited to enrol on a feasibility RCT with embedded qualitative study. The primary outcome will be feasibility; process-related measures will include the number of people eligible, recruited, randomised and withdrawn, along with indicators of exercise programme adherence and acceptability. Secondary physiological, clinical and health-related outcomes including sub-maximal recumbent bike exercise test, active stand test and HRQoL will be measured at 4 and 7 months post-randomisation by researchers blinded to treatment allocation. The PostUraL tachycardia Syndrome Exercise (PULSE) intervention consists of (1) individual assessment; (2) 12-week, once to twice-weekly, supervised out-patient exercise training; (3) behavioural and motivational support; and (4) guided lifestyle physical activity. The control intervention will be best-practice usual care with a single 30-min, one-to-one practitioner appointment, and general advice on safe and effective physical activity. For the embedded qualitative study, participants (*n* = 10 intervention, *n* = 10 control) will be interviewed at baseline and 4 months post-randomisation to assess acceptability and the feasibility of progressing to a definitive trial.

**Discussion:**

There is very little high-quality research investigating exercise rehabilitation for people with POTS. The PULSE study will be the first randomised trial to assess the feasibility of conducting a definitive multicentre RCT testing supervised exercise rehabilitation with behavioural and motivational support, compared to best-practice usual care, for people with POTS.

**Trial registration:**

ISRCTN45323485 registered on 7 April 2020.

## Background

Postural orthostatic tachycardia syndrome (POTS) affects the autonomic nervous system resulting in an abnormal cardiovascular response to upright posture. It is defined as a clinical syndrome that is usually characterised by (1) frequent symptoms that occur with standing such as light-headedness, palpitations, tremulousness, generalised weakness, blurred vision, exercise intolerance and fatigue; (2) an increase in heart rate of ≥ 30 beats per minute when moving from a recumbent to a standing position held for more than 30 s; and (3) the absence of orthostatic hypotension (> 20 mmHg drop in systolic blood pressure) [1]. The pathophysiology of POTS is multi-factorial. Symptoms may result from a multitude of mechanisms, including but not limited to volume depletion, immune dysfunction or autoimmune disease, cardiac and physical deconditioning and the inability of lower limb vasoconstriction to compensate for postural blood volume shifts [2]. A disproportionate autonomic response, aimed at rectifying haemodynamic compromise, results in the hallmark features of prolonged pre-syncope (feeling of being about to faint) and fatigue [1, 3].

POTS can be debilitating; simple activities may result in persistent orthostatic intolerance. This can lead to poor concentration, palpitations, nausea, ‘brain fog’ and exercise intolerance [3] affecting activities of daily living and health-related quality of life (HRQoL) [4]. Hypermobility spectrum disorders (HSD) are a common comorbidity [5]. The ability to attend education, undertake gainful employment and care for dependants can be substantially compromised by POTS. A constellation of symptoms can initiate a negative feedback loop by which enforced inactivity further precipitates orthostatic intolerance, immobility and deconditioning [6, 7].

Treatment for POTS is limited; medical therapies have not been tested in randomised controlled trials (RCTs) [8]. According to expert consensus, non-pharmacological therapy is first-line treatment [1]. Conservative measures include increased fluid and salt intake, compression tights, isometric counter-pressure manoeuvres (e.g. hand gripping and leg crossing/tensing) to prevent fainting, and psychological interventions to help manage chronic illness [1]. Exercise training is also advocated as an essential component of POTS treatment [1]. However, this is based on only two small studies, one of which excluded people with HSD [9] and one which investigated orthostatic intolerance in military recruits [10]. These studies demonstrated some physiological improvement (increased left ventricular mass/blood volume and decreased standing heart rate), reduced symptoms and improved HRQoL. More recently, a prospective observational study (*n* = 251) enrolled people with POTS on 3 months of unsupervised community exercise [8]. A total of 103 people completed the treatment, of which 73 no longer met the diagnostic criteria for POTS, and HRQoL was improved. However, the study was limited by the lack of a control group, and poor individualisation and supervision of exercise, leading to 60% attrition.

### Rationale for a trial

Supervised exercise rehabilitation may be an effective therapy for POTS, with resultant clinical and psychosocial benefit. Observational studies, whilst not definitive, suggest that symptoms and psychosocial morbidity may improve [8]. Based on this preliminary evidence, it is important to investigate whether or not people with POTS can benefit from supervised exercise rehabilitation. The broad spectrum of physical ability, symptoms and comorbidities requires that this be initially undertaken within well-designed clinical trials that take account of previous research limitations. The first step is to investigate the feasibility of conducting a multi-centre RCT testing a comprehensive exercise rehabilitation intervention, compared to best-practice usual care, for people with POTS.

## Methods/design

### Aim

The aim of the PostUraL tachycardia Syndrome Exercise (PULSE) study is to assess the feasibility of conducting a multi-centre RCT testing a supervised exercise rehabilitation intervention with behavioural and motivational support (PULSE intervention) compared to best-practice usual care for people with POTS.

### Objectives

#### Undertake a feasibility RCT:

Sixty-two participants (*n* = 31 intervention, *n* = 31 control) will be randomised to the PULSE intervention or control. Process-related measures will include eligibility, recruitment, uptake and intervention adherence. Physiological, clinical and health-related measures will include a sub-maximal graded recumbent bike exercise test, active stand test and HRQoL.

#### Conduct a qualitative analysis:

To explore perceptions, opinions, acceptability and experiences of trial procedures, the PULSE intervention and outcome measures, interviews will be conducted with a sample of participants, drop-outs, and those who declined participation.

### Study design and setting

**Fig. 1:**
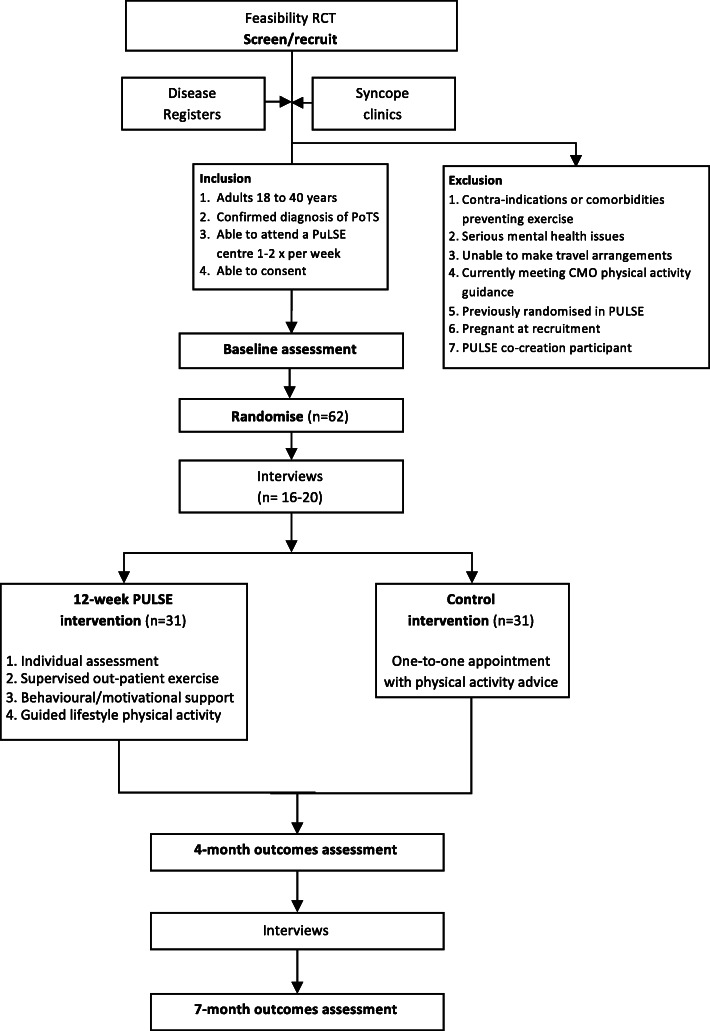
Study flow chart

PULSE is a two-arm feasibility RCT with an embedded qualitative study (Fig. 1). Recruiting simultaneously from two NHS POTS clinics in England (University Hospitals Coventry & Warwickshire (UHCW) NHS Trust and Imperial College Healthcare NHS Trust), participants will be randomised to the intervention or control arms in a ratio of 1:1, stratified by NHS Trust. Table 1 summarises study methods and design in accordance with the World Health Organization (WHO) Trial Registration Data Set.

**Table 1:** World Health Organization trial registration data set

Data category	Information
Primary registry and trial identifying number	ISRCTN45323485
Date of registration in primary registry	7 April 2020
Secondary identifying numbers	REC reference: 20/EM/0077 BHF reference: PG/19/22/34203
Source(s) of monetary or material support	British Heart Foundation Project Grant
Primary sponsor	UHCW NHS Trust University Hospital Clifford Bridge Rd., Coventry CV2 2DX Tel: 02476 966195 Email: R&DSponsorship@uhcw.nhs.uk
Secondary sponsor(s)	Coventry University Richard Crossman Building Jordon Well Coventry University CV1 5RW Email: cdu141@coventry.ac.uk
Contact for public queries	Coventry University Richard Crossman Building Jordon Well Coventry University CV1 5RW Email: cdu141@coventry.ac.uk
Contact for scientific queries	Dr Gordon McGregor Coventry University/UHCW NHS Trust Tel: 024 76150285 Email: gordon.mcgregor@coventry.ac.uk
Public title	Supervised exercise rehabilitation for people with postural tachycardia syndrome
Scientific title	PostUraL tachycardia Syndrome Exercise (PULSE): a randomised controlled feasibility study
Countries of recruitment	England
Health condition(s) or problem(s) studied	Postural orthostatic tachycardia syndrome
Intervention(s)	Intervention group: (1) individual assessment, (2) supervised out-patient exercise programme, (3) behavioural and motivational support, (4) guided lifestyle physical activity. Control intervention: best-practice usual care
Key inclusion and exclusion criteria	Inclusion: adults (18–40 years) with confirmed diagnosis of POTS and attending syncope out-patient clinics; able to attend a PULSE centre; able to provide informed consent. Exclusion: absolute contraindications to exercise; currently achieving CMO physical activity guidelines; mental health issue preventing engagement with trial procedures; pregnant at time of recruitment; previous randomisation in PULSE; unable to attend a PULSE centre; took part in PULSE intervention co-creation.
Study type	Type: feasibility, interventional, two-centre Allocation: randomised Assignment: parallel Masking: outcomes assessors, chief investigator, statistician
Date of first enrolment	TBC
Target sample size	62
Recruitment status	Ready to start recruitment (on hold due to Covid-19)
Primary outcome(s)	Feasibility and process indicators: number of patients screened, eligible, recruited, randomised, withdrawn and retained; adherence to exercise rehabilitation programme; length of time to complete each outcome assessment and the whole outcome assessment appointment; willingness of participants to join non-POTS specific exercise rehabilitation programmes; physiological, clinical, patient-reported outcomes to identify a primary outcome for a definitive trial; acceptability of the interventions and the trial (qualitative interviews)
Key secondary outcomes	At 4 and 7 months: exercise capacity—graded recumbent cycle ergometer test; autonomic function—increase in heart rate from supine to 10-min stand; symptom burden—COMPASS 31 dysautonomia scale and fatigue severity scale; HRQoL—EQ-5D-5L; self-efficacy—general self-efficacy scale; exercise tolerability—continuous heart rate monitoring and symptoms during exercise; adverse events. At baseline and 7 months: semi-structured interviews with participants

### Eligibility criteria

People with POTS meeting the study inclusion criteria are eligible to participate (Table 2).

**Table 2:** Inclusion and exclusion criteria

Inclusion criteria	Exclusion criteria
▪ Adults 18 to 40 years of age ▪ Confirmed diagnosis of POTS [1] and currently attending specialist out-patient clinics ▪ Able to attend a PULSE centre 1–2 times/week for 8–12 weeks for exercise training ▪ Able to provide informed consent	▪ Absolute contraindications to exercise as per international clinical guidelines [11, 12] ▪ Any serious mental health/cognitive issue that will prevent engagement with study procedures or increase the risk of exercise complications ▪ Unable to make suitable travel arrangements ▪ Currently undertaking structured exercise/physical activity equivalent to the Chief Medical Officer (CMO) guidelines (150 min moderate exercise per week or 60 min vigorous per week) ▪ Previous randomisation in the present trial ▪ Pregnancy ▪ Taken part in co-creation workshops to design the PULSE intervention/study

### Participant identification, recruitment and informed consent

Physicians, arrhythmia nurse specialists or other clinical staff involved in the care of people with POTS will identify eligible participants from two sources: (1) specialist nurse/medical POTS out-patient clinics, (2) local secondary care disease registers. Participant information leaflets will be provided in person during an out-patient clinic appointment or will be sent in the post with a request to return an expression of interest form. Once interest and willingness to be contacted have been confirmed, participants will be contacted by a member of their clinical team and invited to attend a baseline assessment appointment, at which eligibility will be reassessed and informed consent taken. The PULSE study requires different levels of consent: (1) to take part in the trial; (2) for medical notes to be reviewed by designated individuals and personal identifiable information to be securely stored; and (3) to allow telephone contact between the trial team and the participant or designated next of kin.

### Randomisation, allocation concealment and blinding

**Fig. 2:**
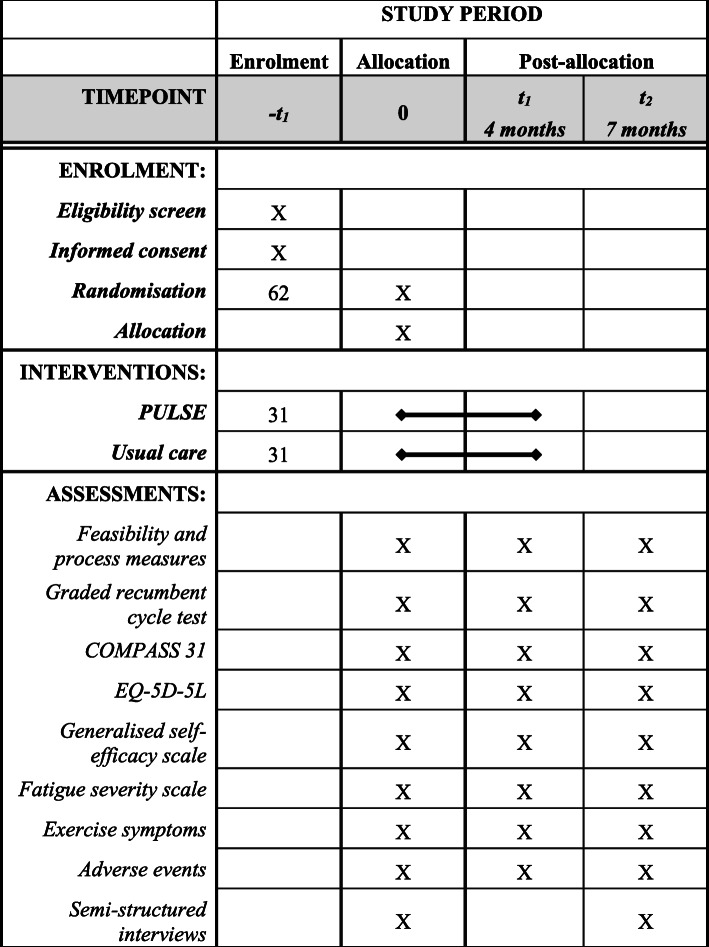
Schedule of enrolment, interventions and assessments

After all baseline assessments have been completed, participants will be randomised on a 1:1 basis to intervention or control, stratified by centre (UHCW NHS Trust or Imperial College Healthcare NHS Trust) (Fig. 2). Randomisation will be performed, and allocation concealment maintained, using an online validated randomisation sequence generator, as part of an electronic data capture system (Castor). Only PULSE practitioners, who will be delivering either the control or active interventions, will be informed of group allocation, and only after all baseline measures are complete. Participants and practitioners cannot be blinded to group allocation. PULSE practitioners will deliver both the active and control interventions, so will be aware of treatment allocation after randomisation. Outcome assessment staff, the chief investigator (CI) and the trial statistician will be blinded to group allocation, until statistical analysis is complete.

### Interventions

#### The PULSE intervention

To ensure applicability and acceptability further to receipt of study grant funding, the intervention was refined and finalised using co-production methods, in accordance with INVOLVE [13]. A detailed account of this process will be available in an intervention development publication. Briefly, two workshops with patients, public and stakeholders were led by experienced facilitators. During the first workshop, proposed outcome measures and the PULSE intervention were presented to the group and discussed from all stakeholders’ perspectives. Interim work was then undertaken by the research team to refine the intervention components and outcome measures prior to workshop two during which the protocol was finalised and implementation and delivery were discussed.

POTS patients and stakeholders (family, nursing, psychology, exercise physiology, physiotherapy, general practice, POTS UK) attended the meetings to ensure that contributions were correctly interpreted [14]. Co-production techniques allowed these multiple stakeholders to work towards a common goal [13]. It was a flexible and adaptable process, using multiple communication strategies, to ensure inclusion of people who were affected differently by POTS. The process was iterative, with each session informing the next, until a solution was achieved. Researcher notes were analysed thematically, thus informing the refinement and implementation of the PULSE intervention and study [15].

##### Format:

To ensure external validity to the NHS out-patient/community outreach setting, the PULSE intervention is based on existing UK cardio-pulmonary rehabilitation guidelines and service design [16–18].

##### Programme design:

Participants randomised to the PULSE intervention will either (1) access existing cardiopulmonary rehabilitation programmes, i.e. they will exercise with people with a range of cardiopulmonary conditions, as well as other trial participants randomised to the PULSE intervention, or (2) they will attend dedicated sessions for people with POTS. To encourage peer support, every effort will be made to ensure there are at least two to three PULSE participants exercising at the same centre at the same time. The PULSE intervention has four components:

#### Component 1: individual assessment and exercise familiarisation

##### Individual assessment:

Participants will undergo a 1-h, one-to-one appointment with a PULSE ‘practitioner’ (clinical exercise physiologist), to assess and record medical and physical activity history and medication and to discuss goals, expectations and any concerns.

##### Exercise prescription:

There are no accepted guidelines for exercise training in POTS. The intervention will be individualised and is based on existing evidence, patient and public involvement (PPI) and co-production sessions and the centres’ expertise in the provision of exercise for clinical populations. As such, the PULSE practitioner will prescribe a safe and effective individualised exercise programme [11, 17] based on clinical information, data from graded, sub-maximal cycle ergometry and active stand tests and patient-centred goal setting.

##### Familiarisation sessions:

Exercise instruction will be provided on an individual basis during two one-on-one familiarisation sessions in the first week of the programme, and reinforced throughout, by PULSE practitioners and clinical staff. Familiarisation sessions, conducted within the cardiopulmonary rehabilitation programmes, will enable participants to build confidence, whilst PULSE practitioners refine and optimise the exercise prescription. Behavioural and motivational support will also be introduced during these sessions.

#### Component 2: supervised out-patient exercise programmes

For the first 6 (of 12) weeks, exercise will be undertaken during one to two sessions per week solely in a controlled gym environment with carefully staged progression in response to participant tolerance and symptoms. For those that are able, moderate intensity dynamic cardiovascular exercise will be prescribed. In addition, ‘functional fitness training’ will aim to improve orthostatic tolerance and general musculoskeletal deconditioning. This type of training is targeted specifically at the components of physical fitness required for activities of daily living, making use of multi-plane motion, to improve agility, coordination, proprioception, balance and functional strength.

##### Prescribed exercise:

Exercise will be versatile and individualised, incorporating cardiovascular and functional resistance training components. Interval or continuous cardiovascular exercise will be performed at a tolerable intensity (regulated with rating of perceived exertion [RPE] and symptoms) for a manageable time, focusing initially on exercises in the recumbent or semi-recumbent position (e.g. rowing machine, recumbent cycle ergometer) to minimise orthostatic tachycardia. Gentle warm-up and cool down will be performed in the manner best suited to individual symptoms. Exercise intensity and time will be gradually increased, and upright exercise introduced as tolerated.

#### Component 3: behavioural and motivational support

Comprehensive behavioural change and motivational strategies to improve adherence and compliance to exercise will be incorporated into the PULSE intervention. Every second week, before or after their supervised exercise session, participants will receive a one-to-one, 30-min behavioural and motivational session delivered by a trained PULSE practitioner, with the aim of improving short- and long-term adherence to exercise.

The PULSE intervention will be based on a cognitive behavioural framework including self-efficacy and self-management principles with behaviour change theory [19]. In particular, the COM-B model of behaviour change (‘capability’, ‘opportunity’, ‘motivation’ and ‘behaviour’) will be applied to the content and delivery of the behavioural sessions. Capability relates to increasing confidence through supervised exercise practice and exploring participants’ own health beliefs and perceived barriers which may prevent them from progressing with the intervention. For example, quite often people with POTS may avoid certain activities and movement due to a fear of aggravating symptoms [20]. Opportunity, both internal and external to the individual, will be explored through beliefs and thoughts in relation to knowledge and acceptance of POTS, including principles of self-management and skills of living with what is sometimes termed ‘the invisible condition’. Access to material and information covered in the sessions will be ensured by producing a participant handbook to allow consolidation of learning and reflection in between sessions. Motivation will also be key to sustained behaviour change for people with POTS. This will be addressed with planning, goal setting and exploration of expectations, with opportunity for self-reflection and discussion of progress, setbacks and overcoming obstacles [21]. This will be combined with the theory of planned behaviour [22, 23] to explore participant’s perceived control, intention and motivation to adhere to the PULSE intervention.

The PULSE practitioners will be trained to use open questions and motivational interviewing to assess participants’ current beliefs and encourage behaviour change. In accordance with existing literature, it will be important to manage expectations. Previous work in POTS has indicated that symptoms may worsen for 4–6 weeks before a lasting benefit is gained [21].

#### Component 4: lifestyle physical activity

Half-way through the supervised exercise intervention, thus after 6 weeks of centre-based exercise has been safely completed, and/or individual exercise tolerance has been evaluated, participants will be encouraged to undertake self-directed, lifestyle physical activity at home in addition to the supervised sessions. This will help ensure that exercise is performed every other day. Activities such as swimming, walking and cycling will be encouraged for those in whom it is appropriate.

#### Control intervention: best practice usual care

Participants in the control arm will be provided with freely available advice on lifestyle physical activity (POTS UK website) [24] during a one-to-one session, lasting approximately 30 min, with a PULSE practitioner. They will not receive any further input during the control intervention period. Participants will be permitted to continue with any current physical activity but will not receive supervised exercise training or behavioural and motivational sessions from PULSE practitioners.

### Safety

The PULSE intervention will be delivered in cardiopulmonary rehabilitation units with qualified staff and appropriate emergency equipment. Lifestyle physical activity will be lower intensity and replicate exercises performed in the gym under supervision. Intervention practitioners will be clinical exercise physiologists, experienced in assessment, prescription and delivery of exercise in clinical populations and trained in the standardised delivery of the PULSE intervention. Additional training in exercise modification to avoid joint subluxation and dislocation in hypermobile participants will be provided by a specialist physiotherapist.

### Primary outcome

The primary outcome will be overall feasibility and process-related measures:

- Number of patients screened, eligible, recruited, randomised, withdrawn and retained.
- Adherence to exercise rehabilitation programme—number of supervised exercise and lifestyle physical activity sessions completed over 12 weeks.
- Length of time to complete each outcome measure and the whole outcome assessment appointment.
- Willingness of participants to join non-POTS specific exercise rehabilitation programmes (i.e. standard cardiopulmonary rehabilitation programmes). Participant preference will be recorded, and uptake to each programme model monitored.
- Physiological, clinical, patient-reported outcomes to confirm/identify a primary outcome for a definitive trial.
- Acceptability of the interventions and the trial (embedded qualitative study).

### Secondary outcomes

Physiological, clinical and patient-reported outcomes will be assessed during study visits at baseline, and post-intervention at 4 and 7 months post-randomisation (Table 2).

Exercise capacity will be measured with a graded sub-maximal recumbent cycle ergometer assessment [11]. This will be a simple test (maximum 10 min) involving gradual increments in workload every minute. Workload increments and test termination will be determined by symptoms, heart rate, blood pressure and RPE responses which will be recorded throughout. The increase in heart rate from supine to 10-min stand test (active stand test) will be conducted as per clinical practice [25]. This is a commonly used diagnostic test for POTS, measuring the heart rate response to 10 min (or however long is manageable dependant on symptoms) of standing. It will be performed only as an outcome measure in this trial, not for diagnostic purposes.

The symptom burden of POTS will be measured with the Composite Autonomic Symptom Score (COMPASS 31) dysautonomia scale [26]. This is a self-rating questionnaire evaluating six domains of autonomic function: orthostatic intolerance, vasomotor, secretomotor, gastrointestinal, bladder and pupillomotor domains. In addition to domain scores, an overall total score from 0 to 100 can be computed, with a higher score indicating a greater disease burden. Health-related quality of life will be evaluated with the EQ-5D-5L [27], a validated, generic HRQoL measure consisting of five dimensions, each with five levels of response. It has good test-retest reliability, is simple to use and gives a single preference-based index value for health status. The Fatigue Severity Scale [28], a validated nine-item questionnaire, will be used to assess disabling fatigue. Each item is rated on a seven-point scale, from strongly disagree to strongly agree. A total score is derived from all nine questions; a higher score indicates a greater impact of fatigue on everyday activities. Self-efficacy will be measured with the generalised self-efficacy scale [29] which is a 10-item psychometric scale designed to assess optimistic self-beliefs to cope with a variety of difficult demands in life. A single score between 10 and 40 is generated; greater self-efficacy is represented by a higher score.

To evaluate the participants’ response to exercise sessions and assessments, any POTS-related or associated symptoms will be recorded. Adverse and serious adverse events will be documented and reported to the trial management group (TMG) in accordance with the principles of good clinical practice (GCP).

### Sample size

This is a feasibility RCT to assess recruitment, uptake, adherence, acceptability and clinical, physiological and health-related outcomes. Thus, the sample size is not based on a power calculation. Nevertheless, the aim is to estimate any possible effect of the change of heart rate from supine to 10-min stand between the two treatment arms to inform a future definitive RCT. The sample size is determined using the confidence interval approach to provide a given level of precision. From a single-arm study investigating a 3-month community exercise programme in a similar population [8], the mean increase in heart rate from supine to 10-min stand at baseline was 46 ± 17 bpm and post-intervention was 23 ± 14 bpm. For the purpose of this trial, the standard deviation (SD) is assumed to be the same for both treatment arms, and the SD of the difference of the change is 17 bpm. Therefore, the total sample size required to obtain a 95% confidence interval width of 20 bpm is 46 participants [30]. Assuming a drop-out rate of 25%, the total sample size required is 62 participants (31 per treatment arm). Participants will be randomised equally to the intervention or usual care arms (i.e. 1:1 ratio).

### Data analysis

Participant demographics and physiological, clinical and patient-reported outcomes will be summarised by treatment arms as mean and SD, median and interquartile range (for continuous data) or frequency and percentage (for categorical data) at baseline, and 4 and 7 months post-randomisation. The primary outcome is feasibility; therefore, process indicators relating to recruitment and intervention delivery will be assessed. Descriptive statistics will be presented by recruitment centre and treatment arm. The difference in outcomes between treatment arms at 4 and 7 months post-randomisation and the difference of the change from baseline to 4 months post-randomisation between arms will be computed. All estimates will be reported with a 95% confidence interval. No formal hypothesis testing will be performed.

All data will be analysed and reported in accordance with the CONSORT statement. All primary analyses are planned on an intention-to-treat basis (i.e. data will be analysed according to the treatment arm the participant was originally allocated to, irrespective of what treatment they actually received) with a secondary per protocol analysis.

### Embedded qualitative study

Semi-structured interviews, conducted by an experienced qualitative researcher, with approximately *n* = 10 intervention group and *n* = 10 usual care, at baseline and 4 months post-randomisation (same participants at both time-points), will be digitally recorded, pseudonymised and transcribed verbatim. Data will be analysed using the framework method [31], broadly as follows:

- Data familiarisation: reading of complete interview transcripts, listening to original audio recordings and use of field notes;
- Identifying a thematic framework: key issues, concepts and themes will be identified, and an index of codes developed;
- Indexing: the index generated through identification of the thematic framework will be applied to all data;
- Charting: a summary of each passage of text will be transferred into a chart to allow more overall and abstract consideration of index codes across the data set and by each individual;
- Mapping and interpretation: understanding the meaning of key themes, dimensions and the broad overall picture of the data and identifying and understanding the typical associations between themes and dimensions.

The charting process will provide an opportunity to code data from numerous perspectives. The computer package NVivo will be used to organise the analysis. The findings of the qualitative analyses will be reported as a separate chapter in the final report but will also be incorporated in the discussion to bring together a synthesis of all the results, thus helping to explore and explain the overall ‘value’ of the interventions. Quantitative and qualitative data will be integrated using a mixed methods matrix where quantitative responses can be compared to interview data and recorded on a matrix [32].

### Transition to a definitive trial

The ADePT (A Process for Decision-making after Pilot and Feasibility Trials) framework [33] will be used to identify and examine issues and problems methodically, and to appraise and find appropriate solutions to inform the decision-making process to transition from this two-centre feasibility trial to a definitive multi-centre RCT.

### Data collection and management

Personal data collected during the study will be handled and stored in accordance with General Data Protection Regulation (GDPR). Personal identifying information will be stored at each site for follow-up purposes. Disclosure of confidential information will only be considered if there is an issue which may jeopardise the safety of the participant or another person, in accordance with local standard operating procedures (SOPs) and the UK regulatory framework. An online validated, GCP compliant, electronic data capture system (Castor) will be used by researchers at each site to record and store study data. All data will be pseudonymised after the collection of baseline demographics for each participant. Participants will be assigned a unique study ID, which will be used to identify all documents associated with that participant for the duration of the study. In line with participant consent, direct access to source data/documents will be granted to authorised representatives from the sponsor, host institution and the regulatory authorities, to permit study-related monitoring, audits and inspections.

### Trial monitoring

The trial management group (TMG), chaired by the CI, and consisting of project management staff, lay representatives, co-investigators, the trial statistician and sponsor representatives, will meet monthly for the duration of the project. For this small feasibility RCT, there will be no independent data monitoring committee or trial steering committee; these functions will be completed by the TMG.

### Adverse event management

An adverse event (AE) will be defined as any untoward medical occurrence involving a participant, which does not necessarily have a causal relationship with the intervention or trial. Expected AEs for the PULSE study that will be recorded but not reported include pre-syncope, muscle and joint stiffness, soreness and dislocation and tiredness and fatigue. Any unexpected AEs related to PULSE will be recorded and reported to the TMG. Serious adverse events (SAEs) that have no causal relationship with PULSE will not be reported to oversight committees. Causality and expectedness will be confirmed by the CI with clinician support. SAEs deemed to be unexpected and possibly, probably or definitely related to PULSE will be notified to the Research Ethics Committee (REC) within 15 days. All AEs and SAEs will be recorded within 24 h of the investigator being made aware.

### Patient and public involvement

The PULSE intervention components were initially developed based on existing evidence, previous clinical work at our centres and extensive patient and public involvement (PPI). Eighteen people with POTS participated in teleconference consultation prior to the grant funding application. The intervention was further developed and refined during two co-creation workshops with multiple stakeholders (as outlined in more detail above). Lay representatives will sit on the TMG and will be invited to contribute to dissemination and reporting on completion of the study.

## Discussion

Postural orthostatic tachycardia syndrome can be a debilitating condition, with pre-syncope and fatigue, amongst other symptoms, significantly impacting on activities of daily living and HRQoL. Whilst prevalence is estimated to be between 0.2 and 1%, it is increasingly recognised that POTS is commonly misdiagnosed, thus prevalence is likely to be much higher. In the absence of effective and appropriately tested medical and pharmacological therapies, lifestyle interventions have the potential to improve symptoms and help manage this long-term condition.

Exercise rehabilitation is not routinely offered to people with POTS as it has not been evaluated in well-designed clinical trials. Preliminary reports from non-randomised controlled studies indicate that exercise rehabilitation programmes, as provided almost universally for people with long-term cardiac and pulmonary conditions, may be beneficial for people with POTS. The PULSE study will be the first to assess the feasibility of an exercise intervention with behavioural and motivational support for people with POTS, to inform whether or not a future multi-centre RCT investigating clinical and cost-effectiveness can be delivered in the NHS. The feasibility of a supervised exercise programme, a behavioural and motivational support intervention, and a number of important clinical, physiological and patient-reported outcome measures, will be evaluated within the framework of a feasibility RCT. If feasibility is proven, a definitive multi-centre RCT should be conducted in the future.

## Trial status

All approvals are in place, and the trial is ready to begin recruitment. Currently, all trial activities have been temporarily suspended due to the COVID-19 pandemic.

## Data Availability

The datasets used and/or analysed during the current study will be available from the corresponding author on reasonable request.

## Abbreviations

ADePT: A Process for Decision-making after Pilot and Feasibility Trials
AE: Adverse event
CMO: Chief Medical Officer
CI: Chief investigator
COMPASS-31: Composite autonomic symptom score
CONSORT: Consolidated Standards of Reporting Trials
FSS: Fatigue Severity Scale
GCP: Good Clinical Practice
GDPR: General Data Protection Regulation
HADS: Hospital Anxiety and Depression Scale
HRQoL: Health-related quality of life
HSD: Hypermobility spectrum disorders
IRAS: Integrated Research Application System
ISRCTN: International Standard Randomised Controlled Trial Number
PI: Principle investigator
POTS: Postural orthostatic tachycardia syndrome
PPI: Patient and public involvement
RCT: Randomised controlled trial
REC: Research Ethics Committee
R&D: Research and development
RPE: Rating of perceived exertion
SAE: Serious adverse event
SAP: Statistical analysis plan
SD: Standard deviation
SOP: Standard operating procedure
UHCW: University Hospitals Coventry & Warwickshire
WHO: World Health Organization
